# Cervical collagen and biomechanical strength in non-pregnant women with a history of cervical insufficiency

**DOI:** 10.1186/1477-7827-8-92

**Published:** 2010-07-30

**Authors:** Birgitte S Oxlund, Gitte Ørtoft, Annemarie Brüel, Carl Christian Danielsen, Hans Oxlund, Niels Uldbjerg

**Affiliations:** 1 Department of Obstetrics and Gynecology, Aarhus University Hospital, Skejby, DK-8200 Aarhus N, Denmark; 2 Institute of Anatomy, Aarhus University, DK-8000 Aarhus C, Denmark

## Abstract

**Background:**

It has been suggested that cervical insufficiency (CI) is characterized by a "muscular cervix" with low collagen and high smooth muscle concentrations also in the non-pregnant state. Therefore, the aim of this study was to investigate the biomechanical properties, collagen concentration, smooth muscle cell density, and collagen fiber orientation in cervical biopsies from non-pregnant women with a history of CI.

**Methods:**

Cervical punch biopsies (2 × 15 mm) were obtained from 57 normal non-pregnant women and 22 women with a history of CI. Biomechanical tensile testing was performed, and collagen content was determined by hydroxyproline quantification. Histomorphometry was used to determine the volume densities of extracellular matrix and smooth muscle cells from the distal to the proximal part of each sample. Smooth muscle cells were identified using immunohistoche-mistry. Finally, collagen fiber orientation was investigated. Data are given as mean +/- SD.

**Results:**

Collagen concentration was lower in the CI group (58.6 +/- 8.8%) compared with the control group (62.2 +/- 6.6%) (p = 0.033). However, when data were adjusted for age and parity, no difference in collagen concentration was found between the two groups. Maximum load of the specimens did not differ between the groups (p = 0.78). The tensile strength of cervical collagen, i.e. maximum load normalized per unit collagen (mg of collagen per mm of specimen length), was increased in the CI group compared with controls (p = 0.033). No differences in the volume density of extracellular matrix or smooth muscle cells were found between the two groups. Fibers not oriented in the plane of sectioning were increased in CI patients compared with controls.

**Conclusions:**

Cervical insufficiency does not appear to be associated with a constitutionally low collagen concentration or collagen of inferior mechanical quality. Furthermore, the hypothesis that a "muscular cervix" with an abundance of smooth muscle cells contributes to the development of cervical insufficiency is not supported by the present study.

## Background

Various definitions of cervical insufficiency (CI) have been suggested. Of those, the most prominent include the following: 1) an inability of the uterine cervix to retain a pregnancy in the absence of uterine contractions [1]; and 2) a painless, progressive dilatation and effacement of the cervix that may lead to second trimester abortions or preterm delivery [2]. The latter may be the more clinically applicable. However, CI remains a diagnosis of exclusion because the pathophysiology of CI remains unknown. Furthermore, CI might be seen as an extended biological continuum with degrees of cervical competence [3], replacing the traditional dichotomous view of cervical competence as being present or absent. However, it is well established that changes in connective tissue are important in the process of cervical ripening and remodeling [4] as the normal cervix is dominated by connective tissue rich in collagen, with only 15% muscle cells [5].

One hypothesis describes CI as a pregnancy-induced preterm cervical ripening involving increased inflammatory response, characterized by the up regulation of cytokines, prostaglandins and matrix metalloproteinases [6,7]. This hypothesis is supported by a study showing that polymorphisms in the promoter region of the interleukin 10 (IL-10) gene are more common in women with CI than controls [8].

Another hypothesis explains CI as a constitutional defect in the cervical tissue, present in both the non-pregnant and pregnant states. A general connective tissue defect is seen in patients with Ehlers-Danlos syndrome [9] (Classical EDS, which results from mutations in the COL5A1 gene [10,11]), who deliver preterm due to CI or as polymorphisms in the collagen 1A1 gene (*COL1A1*) found in CI patients [12]. Furthermore, hypotheses on localized cervical defects such as decreased collagen concentration [13-15], elastic fiber content [16] or increased smooth muscle cells (the "muscular cervix" [17,18]) have been suggested.

The aim of the present study was to investigate the hypothesis that CI is caused by a constitutional defect in the cervical tissue also present in a non-pregnant state. We hypothesize that non-pregnant women with a history of CI have decreased cervical collagen concentration, decreased biomechanical strength of the cervical tissue and increased smooth muscle cells compared with women who delivered at term.

## Methods

This study was approved by the Local Research Ethical Committee (Region of Midtjylland, journal number: 20040195) and conducted in accordance with the Declaration of Helsinki 2008. In the present study the following definition of CI was used: a painless dilatation of the cervix in the second trimester of pregnancy, with no contractions of the uterus and no vaginal bleeding.

### Study population

This case-control study included fifty-seven normal non-pregnant women (Table 1), admitted for sterilization (results on biomechanical properties and collagen concentration within this group was previously published [19]), as well as 22 non-pregnant women with a history of CI (Table 2). Exclusion criteria for controls were as follows: history of preterm delivery, conization, cervical laceration, cervical dysplasia, menopause, and connective tissue disorders. At five hospitals (Aarhus, Hvidovre, Viborg, Aalborg and Randers Hospital), 662 patients were identified by ICD10 diagnosis (cervical incompetence), as well as by the procedure, 'cerclage', or short length of the cervix (< 2.5 cm). 599 patients were excluded due to unfulfilled diagnostic criteria for CI, at least one normal birth, signs of infection, previous conization, cervical dysplasia, twin pregnancy or incomplete history. The remaining 66 patients were contacted, and 22 accepted to participate in the study and were included. The period of time elapsed from last birth (controls) or CI to biopsies were taken as follows: controls, 6 years (range 1/2-19 years); and CI, 2 years (range 1/2-9 years).

**Table 1:** Clinical data on the 57 control women

Patient number	Age	Termination of pregnancy <12 weeks	Spontaneous abortion <16 weeks	Spontaneous abortions 16 to 23+6 weeks	Cesarean section	Parity Number of deliveries
1	49	2	-	-	-	3
2	48	1	-	-	-	3
3	45	-	2	-	-	-
4	36	1	2	-	-	3
5	44	-	-	-	-	2
6	30	-	-	-	-	2
7	33	1	-	-	2	2
8	41	1	1	-	-	2
9	40	2	-	-	-	2
10	33	1	1	-	1	2
11	34	-	1	-	1	3
12	41	1	-	-	-	3
13	42	1	-	-	-	2
14	34	-	-	-	-	2
15	41	2	-	-	-	2
16	30	-	1	-	-	2
17	39	1	-	-	-	2
18	41	-	-	-	-	-
19	40	1	-	-	-	3
20	38	-	1	-	1	2
21	30	-	4	-	-	2
22	41	2	1	-	-	3
23	39	2	-	-	-	2
24	38	-	1	-	-	3
25	41	-	1	-	-	3
26	40	1	-	-	-	1
27	37	-	-	-	-	3
28	41	-	-	1	-	3
29	37	-	-	-	2	2
30	32	-	-	-	-	2
31	44	-	1	-	-	2
32	42	1	-	-	-	4
33	36	1	-	-	-	2
34	42	-	2	-	-	4
35	44	-	3	-	-	3
36	33	2	-	-	2	3
37	31	-	-	-	-	-
38	43	-	-	-	-	1
39	30	-	1	-	3	3
40	31	-	1	-	-	3
41	42	-	-	-	-	2
42	42	-	-	-	1	1
43	46	1	-	-	-	4
44	39	1	-	-	-	3
45	35	2	2	-	1	3
46	38	-	-	-	-	4
47	36	-	-	-	2	2
48	42	1	-	-	-	1
49	45	-	-	-	-	-
50	42	1	-	-	-	3
51	31	1	-	-	-	2
52	34	-	1	-	-	2
53	41	1	1	-	-	3
54	41	-	-	-	1	2
55	30	-	-	-	-	-
56	39	1	1	-	-	3
57	29	-	-	-	-	3

All women had normal pregnancies and deliveries at term

**Table 2:** Clinical data on the 22 non-pregnant women with a history of cervical insufficiency

Patient number	Age	Termination of pregnancy before week 12		Spontaneous abortion <16 weeks		Spontaneous abortions 16 to 23+6 weeks		Births (≥24 weeks) gestational age	
**1**	29		-		-	I	23+	II	27+
**2**	37		-		-		-	I	27+ (CS)
**3**	37		-		-	I	22+	II	37+ (Mc-C)
**4**	39	I	8+	III	Un	II	20+	IV	38+ (Mc-C)
				V	Un	VI	17+	VII	36+ (Mc-C)
**5**	27		-		-	I	22+	II	34+ (Abd-C, CS)
								III	38+ (Abd-C, CS)
**6**	30		-		-		-	I	24+ (CS)
								II	37+ (Mc-C)
								III	36+ (Mc-C, CS)
**7**	36		-	I	7+	III	23+	II	25+
								IV	33+ (Mc-C)
								V	35 (Abd-C, CS)
**8**	33		-	II	15+		-	I	25+
								III	38+ (Mc-C)
**9**	30		-	I	6+	II	21	III	37+ (Abd-C, CS)
								IV	36+ (Abd-C, CS)
**10**	36	I	6+	II	8+	III	19+	IV	24+
								V	36+ (Abd-C, CS)
**11**	31		-	I	12+	II	23+	III	39+ (Mc-C)
**12**	38		-		-	I	22+	II	37+ (Mc-C, CS)
								III	37+ (Mc-C)
**13**	32	II	6+		-		-	I	24+ (CS)
								III	35+ (Mc-C)
**14**	26		-		-	I	21+	II	38+ (McC)
**15**	43		-		-		-	I	25+
								II	36+ (Mc-C)
								III	38+ (Mc-C)
**16**	37	I	7+	II	8+		-	V	36+ (Emer-C week 22)
				III	7+			VI	38+ (Mc-C)
				IV	8+				
**17**	43	I	6+	II	14+	V	23+	VI	39+ (Mc-C)
				III	10+			VII	39+ (Mc-C)
				IV	9+			IX	38+ (Mc-C)
				VIII	13+			X	40+ (Mc-C)
**18**	40	I	6+	III	7+	IV	21+		-
		II	8+	VI	6+	V	16+		
									
									
**19**	29		-	-			-	I	28+ ( Emer-C week 19+4)
**20**	25		-	-			-	I	26+ (CS)
**21**	31		-	-		I	22+	II	37 (Mc-C)
**22**	39		-	I	7+	III	21+	-	
				II	8+				

I - X: The sequence of the pregnancies

8+, 16+ etc: Gestational age in weeks

Mc-C: Elective Mc Donald cerclage

Abd-C: Abdominal cerclage

Emer-C: Emergency cerclage on dilated or very short cervix

CS: Cesarean section

Un: Unknown gestational age

### Tissue collection

Long, narrow biopsies of cervical tissue (approximately 15 × 2 mm) were punched out parallel to the cervical canal, halfway between the external os and the lateral surface of the cervix, with an instrument of external diameter 3 mm (Miltex^®^, Dermal Biopsy Punch, Germany). Three biopsies were obtained from each patient at the 3, 6 and 12 o'clock positions. Hemostasis was secured by compression or if necessary with el-coagulation or a stitch. No complications were observed apart from slight vaginal bleeding. As a single biopsy was obtained from one patient in the control group, it was included in the histological part of the study only and not in the biomechanical testing.

Two biopsies were immersed in Ringer's solution and immediately frozen at -80°C until biomechanical testing, and the third biopsy was divided into a proximal and a distal portion. The proximal portion, approximately 5 mm long, was used for later genetic studies. The distal part including the epithelium, approximately 10 mm long, was immersion fixed in 0.1 M sodium phosphate-buffered 4% formaldehyde, pH 7.0, for 24 hours and stored in 70% ethanol until histological examination.

### Biomechanical analysis

The biomechanical analysis was performed on tissue samples from 56 women. Two biopsies from each patient were analyzed by means of a materials testing machine (Alwetron TCT5, Lorentzen & Wettre, Kista, Sweden). The biopsies were thawed at room temperature, and the epithelium WAS removed using a dissecting microscope. Each sample was immersed in Ringer's solution (pH 7.4) and mounted between two clamps with a jaw space of 4 mm. The tensile strength of specimens was tested by moving the clamps apart with a constant deformation rate (10 mm/min), stretching the sample until breaking while a load-deformation curve was recorded.

From the load-deformation curve the following parameters were derived:

F_max_: maximum load (N); the maximum force used for breaking the specimen.

ε-F_max_: strain at maximum load; the specimen extensibility.

S_max_: maximum load; normalized for collagen (N × mm × mg^-1^).

S'_max_: maximum stiffness; normalized for collagen (N × mm × mg^-1^).

F_max _and ε-F_max _imply the biomechanical characteristics of the specimen, whereas S_max _and S'_max _imply the characteristics of the collagen component.

### Determination of hydroxyproline

After mechanical analysis, the tissue between the clamps was used for hydroxyproline determination. The tissue was defatted in acetone, and after freeze-drying the dry defatted weight (DDW) was determined. The tissue was then hydrolyzed in 6 M HCl for 16 h at 100°C. Subsequently, the hydroxyproline content was measured according to Woessner [20] with modifications as described in [21]. The collagen content was calculated by multiplying the hydroxyproline content by 7.46 [22].

### Immunohistochemistry

Immunohistochemistry was used to detect smooth muscle cells. The tissue biopsies were embedded in paraffin, and 2-μm-thick sections parallel to the long axis were cut and mounted two sections per slide. The sections were deparaffinized and endogenous peroxidase blocked by 0.5% H_2_O_2 _in absolute methanol. In order to reveal antigens, sections were boiled for 10 min in 0.1 mM Tris/HCl and 0.5 mM EGTA, pH 9. Non-specific binding was blocked by 1% BSA (bovine serum albumin). Sections were incubated overnight at 4°C with a primary antibody against smooth muscle actin (1:1600, monoclonal mouse anti-human, M0851, DAKO, Denmark) diluted in PBS supplemented with 0.1% BSA and 0.3% Triton-X100. Negative controls were incubated with mouse serum or IgG1 instead of primary antibody. After washing, the sections were incubated with horseradish peroxidase-conjugated secondary antibody (goat anti-mouse, P0447, DAKO, Denmark), for 1 h at 20°C. The peroxidase was visualized by reaction with 0.05% 3,3'-diaminobenzidine tetrahydrochloride dissolved in PBS with 0.1% H_2_O_2 _before counterstaining with Mayer's Haematoxylin and alcoholic eosin. Sections with muscular arteries were used as positive controls.

### Estimation of the volume density of extracellular matrix (ECM) and muscle cells

For the histomorphometry a modified Olympus BH-2 microscope with a motorized stage was used, combined with a video camera (JAI-2040, Kanagawa, Japan). By means of CAST software (Olympus, Denmark), counting frames were superimposed onto live images of the tissue sections. The fields of view in each section were sampled using systematic, uniformly random sampling (SURS) [23]. From a random starting point, a new field of view with fixed x and y distance from the previous field was sampled by means of a motorized specimen stage. From each patient two immunostained sections were evaluated. The epithelium was used to determine the sample orientation. Each section was divided into three or four 2 mm sites, depending on biopsy length (i.e. sites I-IV: 0-2, 2-4, 4-6, 6-8 mm from the epithelium, respectively).

Only sections with detectable epithelium were included in this analysis (controls: n = 50, CI: n = 14). In each section approximately 36 fields of view were evaluated using a counting grid of 81 points. The number of points hitting ECM (defined as non-cellular components), smooth muscle cells (positive for smooth muscle actin), nuclei associated with the connective tissue and blood vessels (with a visible lumen, vessel wall and at times, also blood cells) were counted. The volume density was evaluated for the whole section (all 57 patients included). For sections with detectable epithelium, individual sites I-IV were evaluated as described above (a reduced number of patients were included due to lack of visible epithelium). Counting took place at a final magnification of ×1263. The evaluation of sections was blinded.

### Determination of collagen orientation

Collagen orientation was determined by microscopy (Olympus BX40) with live video imaging (Nikon DS-Fi1) connected to a monitor (Sony Multiscan G200). Nikon NIS-Elements F 3.00 software was used. Three μm thick sections were cut parallel to the long axis of the biopsies and stained with Picro-Sirius [24]. The sections were divided into two portions, of which the proximal portion, corresponding to the part used for mechanical analysis, was used to determine collagen orientation. The epithelium was used to determine section orientation. Only sections with detectable epithelium were included (controls: n = 42, CI: n = 15). A grid was constructed and physically mounted on the computer monitor. With the longitudinal axis of horizontal sections, collagen fibers at least 27 μm long were divided into three categories based on their orientation: 1) fibers deviating less than ±45° from the longitudinal axis; 2) fibers deviating between 46° and 90° or -46° to -90° from the longitudinal axis; and 3) fibers shorter than 27 μm long (indicating that they are not oriented parallel with the sectioning plane). Two sections from each patient were evaluated at a total magnification of ×1115. For each section, collagen orientation was determined at four points within each of 25 randomly (manually) selected fields of view. As above, the evaluation of the sections was blinded.

### Statistical analyses

Data are given as mean ± SD. Comparisons of parameters between the two groups were performed using Students t-test. If necessary, data were log-transformed to comply with the assumption of the statistical method. Linear regression was performed to describe the relationship between a pair of parameters. Multiple linear regression was used to adjust parameters for age and parity. Repeated Measures ANOVA was performed to compare different sites of the histological sections between groups, whereas Test for trend was used to describe a steadily increase or decrease throughout the biopsy. Differences were considered significant when p < 0.05. The statistical packages SigmaStat 3.5 and STATA intercooled 9 were used.

## Results

### Collagen and biomechanical analyses

**Figure 1:**
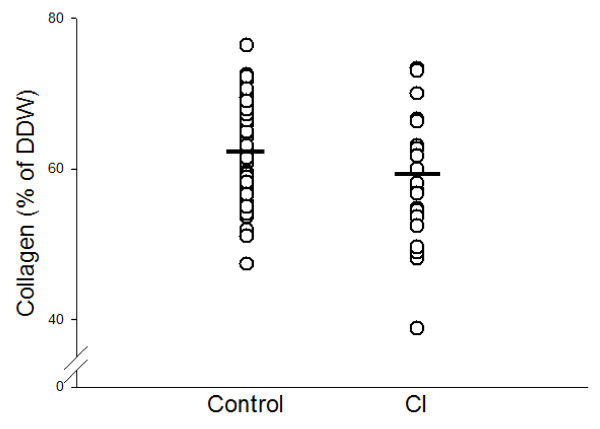
**Collagen concentration in cases and controls**. Collagen concentration (mg collagen/mg dry defatted weight (DDW) ×100) of cervical samples from women with a history of CI and controls (p = 0.033). After adjustment for age and parity by multiple linear regression, no statistically significant difference in collagen concentration was found between groups (p = 0.2).

Collagen concentration was 3.7% lower in the CI-group (58.6 ± 8.8%) (Table 3) compared with the control group (62.2 ± 6.6%) (p = 0.033) (Figure 1). However, as a statistical difference in age and parity was found between the two groups (Table 4), data were adjusted for age and parity. A previous study has shown that the collagen concentration increased 0.5% per year of age and decreased 1.7% per birth [19]. The difference between the two groups was 4.2% (p = 0.02) when adjusted for parity alone, whereas it was 1.6% (p = 0.32) when adjusted for age alone. When adjusted for both age and parity, it was 2.2% and no longer statistically significant (p = 0.2). From these calculations, 43% of the determined difference can be explained due to age, whereas 57% is due to other factors. When collagen concentration in parous women (control group without the 5 nullipara) was compared with the CI group, no difference in collagen concentration was found (p = 0.083).

**Table 3:** Biomechanical and biochemical data

	Control	CI	Control vs. CI	Adjusted values Control vs. CI*
Total number	56	22		
Collagen % (of DDW)	62.2 ± 6.6	58.6 ± 8.8	*p = 0.033*	*p = 0.200*
Collagen (mg/mm)	0.26 ± 0.1	0.22 ± 0.13	*p = 0.059*	*p = 0.835*
F_max _(N)	4.0 ± 1.7	4.1 ± 2.2	*p = 0.778*	*p = 0.335*
S_max _(N × mm × mg^-1^)	15.8 ± 4.4	19.0 ± 6.8	*p = 0.033*	*p < 0.001*
S'_max _(N × mm × mg^-1^)	64.4 ± 21.9	77.3 ± 34.9	*p = 0.096*	*p = 0.008*
ε-F_max_	0.49 ± 0.1	0.49 ± 0.1	*p = 0.914*	*p = 0.212*

Biomechanical and biochemical parameers of cervical samples from women with a history of CI and controls

Data are given as mean ± SD

*Values adjusted for age and parity

**Table 4:** Clinical characteristics of controls and cases

	Control	CI
Total number	57	22
Age (years) mean ± SD	38.3 ± 5.1	34.0 ± 5.3*
Parity (numbers of births) mean ± SD	2.3 ± 1.0	1.7 ± 1**
Spontaneous abortions (<week 16)	35%	45%
Spontaneous abortions 16 to 23+6 weeks	2%	59%
Termination of pregnancy (before week 12)	46%	27%
Cesarean section	19%	41%
Cerclage	0	95%

Percentage of women who experienced one or more i.e. abortions

*p = 0.02 against control

**p = 0.012 against control

Second trimester abortions may have the same impact on the cervical collagen concentration as deliveries. When categorizing the second trimester abortions as deliveries, no difference in collagen concentration was found (adjustment for age, parity and second trimester abortions, p = 0.41).

**Figure 2:**
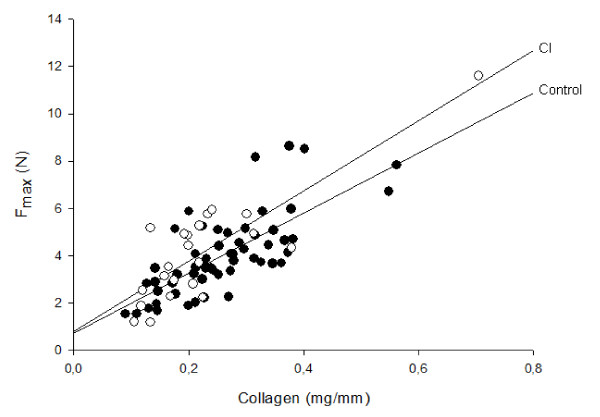
**F_max _versus collagen content**. Maximum load (F_max_) of cervical samples in relation (linear regression) to collagen content mg/mm. **Filled circle**: Control (r = 0.76, p < 0.001). **Open circle**: CI (r = 0.76, p < 0.001) (Result on the control group has been submitted for publication 2009).

To evaluate the hypothesis that CI is caused by a biomechanically weak cervix, the biomechanical properties of cervical tissue samples were investigated. Maximum load (F_max_) was proportional to collagen content both in the control group and the CI group (Figure 2). No difference in maximum load (F_max_) of the biopsies was found between the two groups.

**Figure 3:**
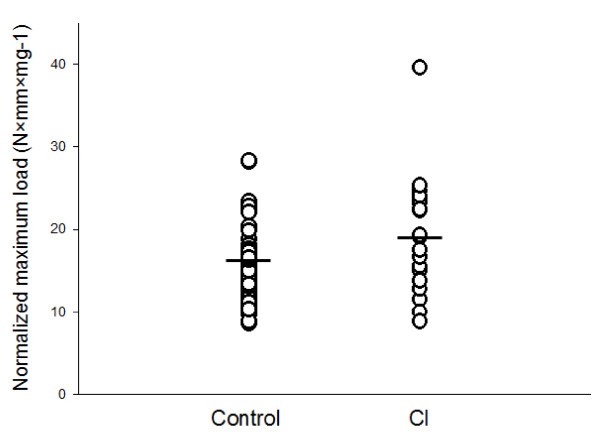
**Normalized maximum load (S_max_) of cases and controls**. S_max_, normalized maximum load, of cervical samples was increased in the CI group compared with controls (p = 0.033). Data were adjusted for age and parity by multiple linear regression (p < 0.001).

In order to describe the tensile strength of cervical collagen, maximum load was divided by collagen content in mg of collagen per mm of specimen length. This normalized maximum load (S_max_) was found to be significantly increased in the CI group compared with controls (Figure 3). Similarly, the normalized maximum stiffness (S'_max_) was determined, but no difference was found between the two groups. However, when values were adjusted for age and parity, the maximum stiffness normalized for collagen was found to be increased in the CI group (p = 0.008; Table 3). No difference in extensibility (ε-F_max_) of the biopsies was found when comparing the two groups. DDW varied considerable, but no significant difference was found between the two groups (CI: 1.90 ± 0.82 mg, controls: 2.13 ± 0.69 mg (p = 0.22)).

### Histomorphometry

To evaluate the hypothesis on the "muscular cervix", the volume density of ECM, smooth muscle cells, connective tissue nuclei, and blood vessel were determined (Table 5); however, no difference was found between the two groups. The sections were divided into site I (distal part, near the epithelium), II, III and IV (proximal part). In both controls and CI the volume density of smooth muscle cells was increased from distal to proximal part of the biopsies (Controls: 8.9 ± 6.7 to 13.9 ± 6.2% (p < 0.001), CI: 5.2 ± 3.0% to 12.0 ± 5.6% (p < 0.001)).

**Table 5:** Histomorphometry

	Control	CI
Number of individuals	57	22
ECM %	76.2 ± 7.1	76.5 ± 8.9
Muscle %	13.3 ± 6.5	11.9 ± 7.8
Connective tissue nuclei %	4.4 ± 1.5	4.8 ± 2.2
Blood vessels %	6.1 ± 3.4	6.7 ± 2.8
ECM/muscle-ratio	8.1 ± 6.5	9.0 ± 5.0

The volume density (%) of cervical samples from women with a history of CI and controls

Data are given as mean ± SD

No significant difference in any of the investigated parameters were found between the two groups

### Collagen fiber orientation

**Figure 4:**
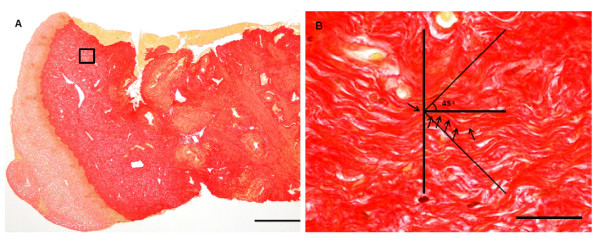
**Collagen fiber orientation**. Picro-Sirius stained sections from the human cervix. (**A**) Longitudinal section of a biopsy including epithelium (Bar: 500 μm); (**B**) collagen fibers in the center of the grid were divided into three categories based on their orientation (longitudinal axis of sections horizontal): **1**) longitudinal or "parallel" fibers (deviating less than ± 45° from the longitudinal axis), **2**) perpendicular fibers, (deviating between 46° and 90° or -46° to -90° from the longitudinal axis) representing circular or radial fibers, and **3**) fibers shorter than 27 μm (not oriented parallel with the sectioning plane) representing circular, radial or wavy longitudinal fibers (arrows point at a longitudinal collagen fiber. Bar: 25 μm).

Collagen fiber orientation was determined in order to evaluate whether the increase in normalized load found in the CI group could be explained from a different orientation of collagen fibers. The percentage of collagen fibers which was not oriented in the plane of sectioning was increased in CI patients (48.5 ± 13.2%) compared with control (40.6 ± 11.6%) (p = 0.033) (Table 6). No differences between the two groups in the percentage of collagen fibers considered "parallel" (less than ± 45°) with (Figure 4A and 4B) or "perpendicular" (between 46° and 90° or -46° to -90°) to the longitudinal axis were found.

**Table 6:** Collagen orientation

	Control n = 42	CI n = 15
Less than ± 45°	36.8 ± 9.6	32.6 ± 7.0
Between 46° and 90° or -46° to -90°	22.4 ± 9.7	19.0 ± 10.7
Fibers not oriented parallel with the sectioning plane (shorter than 27 μm)	40.6 ± 11.6	48.5 ± 13.2*

Orientation of collagen fibers in cervical samples from women with a history of CI and controls. Mean ± SD

*p = 0.033 against control by students t-test

## Discussion

The present study does not support the hypothesis that CI is caused by a constitutional "muscular cervix" [17,18] with low collagen concentration present in both non-pregnant and pregnant states. We found the collagen concentration only marginally and not statistically significantly decreased in non-pregnant women with a history of CI, and neither the biomechanical nor the histomorphometrical examinations supported the hypothesis. The only differences among the CI patients as compared to controls were increased collagen tensile strength and stiffness (S_max _and S'_max _) and increased proportion of collagen fibers not oriented in the plane of sectioning.

Our results on collagen concentration disagree with those of Petersen *et al.*, who found a 15% decreased cervical collagen concentration in non-pregnant CI-women [13], but agree with those of Rechberger *et al.*, who examined early second trimester cervical biopsies from CI-women [25]. We cannot explain these disparities. The muscular cervix hypothesis is based on histological examinations of cervical biopsies taken immediately after delivery [18]. The mean muscle concentration was 9.3 ± 5.9% in 45 controls and 22.3 ± 8.0% in 12 CI-patients. This finding from 1965 has been reproduced, but our results indicate that it cannot be extrapolated to the non-pregnant state.

The biomechanical properties of a tissue are determined not only by the collagen concentration but also by the collagen composition, the cross links, and the presence of other extracellular macromolecules like decorin and versican [26] whereas muscle cells only contribute insignificantly [27]. It is, therefore, interesting that Warren *et al. *[12] found increased frequency of the TT genotype in the COL1A1 gene among CI-patients, a polymorphism associated with abnormal collagen triple-helix composition. We have not studied the COL1A1 gene in our population, but the increased S_max_, which indicates altered collagen properties in CI-patients, could reflect the findings by Warren *et al. *One could also speculate whether such triple-helix abnormalities cause the "coiling" of the collagen fibers and, thus, are not orientated in a specific direction as demonstrated in the CI-patients (Table 5). A polymorphism demonstrated in the TGF-1β gene in CI-patients [12] may also be relevant as the TGF-β pathway is involved in the synthesis of several extracellular macromolecules that interact with collagen.

An explanation for the increased collagen tensile strength (S_max _) and stiffness (S'_max_) in the CI group is not obvious. Differences in collagen fiber orientation might affect the mechanical strength. However, the fraction of collagen fibers oriented "parallel" with the direction of the mechanical testing did not differ between groups (Table 5). Other causes for the difference in collagen strength may reside in differences in the turnover rate of various processes of aging and cross-linking of collagen, or in different interactions between collagen and other extracellular matrix components.

An interesting discovery is the significantly higher portion of collagen fibers, which could not be followed in the plane of sectioning in the CI group, compared with controls. One may speculate that patients with CI have a different collagen fiber orientation. Further investigation on this finding could involve intensive study of collagen fibers in a 3-dimensional reconstruction as collagen fibers are oriented in several directions.

The major strengths of this study are the very restrictive selection of CI patients and the combined application of biomechanical, histological and biochemical analyses. Furthermore, the biopsies were obtained 15 mm deep in the cervical stroma as compared to the superficial biopsies studied by Petersen *et al. *[13] and Rechberger *et al. *[25].

A limitation of the study is the possible bias induced by changed extracellular matrix after previous cerclage treatment of the CI-patients. One could also argue that some relevant anatomical parts of the cervix were not examined. These include the proximal parts of the cervices and parts near the lateral surface of the cervix; however, no difference in smooth muscle cells 15 mm proximal from the epithelium was found between CI and controls.

Before excluding congenital abnormalities as important for the development of CI one could wonder why cervical volumes have never been assessed in women with CI. They might have a relative cervical hypoplasia, which can be identified by ultrasound or MRI examinations. Such studies should be conducted in the near future.

Furthermore, it has been suggested that CI could be part a genetic condition as 25% of CI patients had a first-degree relative with the condition [12]; however, the risk of recall bias was not explained in the given reference.

## Conclusions

In conclusion, cervical insufficiency does not appear to be associated with a constitutionally low cervical collagen concentration or collagen of inferior mechanical quality. Furthermore, the results do not support the hypothesis that a biomechanically weak "muscular cervix" is a cause of CI.

## Competing interests

The authors declare that they have no competing interests.

## Authors' contributions

BO, GØ, AB, HO, CCD and NU contributed to the design of the study. The sample collection was carried out by BO and GØ. Biomechanical and histological analyses were done by BO, AB and CCD. BO, GØ, AB, CCD and NU participated in the data analyses. The manuscript was written by BO with revision by NU, GØ, AB, CCD and HO. All authors have read and approved the final manuscript.
